# Steel ribbed dome structural performance with different node connections and bracing system

**DOI:** 10.1038/s41598-024-64811-0

**Published:** 2024-06-18

**Authors:** Katarzyna Jeleniewicz, Jacek Jaworski, Mariusz Żółtowski, Izabela Uziębło, Anna Stefańska, Saurav Dixit

**Affiliations:** 1 Institute of Civil Engineering, Warsaw University of Life Sciences, Nowoursynowska 159, 02-776 Warsaw, Poland; 2 Faculty of Architecture, University of Ecology and Management in Warsaw, Olszewska 12, 02-792 Warsaw, Poland; 3 Lovely Professional University, Phagwara, Punjab India; 4 Division of Research and Innovation, Uttaranchal University, Dehradun, India; 5 Woxsen School of Business, Woxsen University, Hyderabad, Telangana 502345 India; 6 Centre of Research Impact and Outcome, Chitkara University, Rajpura, 140417 Punjab India

**Keywords:** Engineering, Materials science

## Abstract

The conventional design of steel structure objects relies on a first-order elastic analysis, where the entire object is treated as a set of individual structural elements requiring time-consuming, semi-empirical design calculations. Such an approach leads to inefficient design time and excessive material consumption and may additionally result in designing on the verge of structural safety. The AEC sector's technological and digitization advancement process forces designers to use advanced design methods. Hence, it is necessary to indicate the benefits of using effective optimization. The paper presents a comparative analysis of steel domes using two design approaches: traditional first-order analysis and an advanced second-order analysis. The latter method considers the influence of structural deformation on the magnitude of internal forces. Eight models were developed, varying in terms of the connection's stiffness. The work results identify the differences between the two selected design approaches and present opportunities for further structural performance of steel structures.

## Introduction

A dome is a closed vault shaped like a bowl, most often over a room with a circular plan. It arises through the rotation of a curve about a central axis. Domes are one of the oldest structural roof forms used in architecture. Domes are particularly interesting to engineers as they achieve maximum space with relatively small cross-sections of structural elements. Moreover, because of the aesthetic potential, the structure has a valuable architectural aspect. As new materials were introduced into the Construction Sector, new interpretations of domes were visible in architectural design, from stones, concrete, wood, and steel to many contemporary pavilion materials such as bamboo or 3D printing materials^1^. New technological possibilities, such as rapid prototyping and form finding, bring those structures back to designing as one of the most rigid and bearing the span at the long span without internal supports. The bionic approach to parametric designing improves the ability for more in-depth structural analysis using different software^2^. From the point of view of building mechanics, domes with a steel load-bearing structure are double-curved bar shells. Designing such a structure requires the development of a computational model in which appropriate geometrical, material, and structural assumptions are made and whose task is to reflect the actual behavior of the structure under the influence of loads acting on it. The traditional design of this type of object is based on the first-order elastic analysis and the principle of superposition, i.e. treating the object as a set of individual structural elements^3^. A linear-elastic model of the material and a geometrically linear model of the structure are adopted, applying the system stiffening principle. Such an approach does not take advantage of the current level of technological advancement and digitization of processes that are taking place in the field of construction.

More advanced methods take into account geometric and material non-linearities of structural elements and imperfections, which results in taking into account the interactions between structural elements^4–6^. They are based on the most accurate reflection of the distribution of forces and moments in the structure, including the redistribution of these forces^7,8^. A geometrically non-linear model applies here, in which the influence of structure deformation on the magnitude of internal forces (second-order effects) is considered. According to Eurocode 3^9^, we should test the structure's sensitivity to second-order effects each time we start designing. This susceptibility can be measured using the critical load multiplier, which is the quotient of the critical load value (load corresponding to the global form of elastic instability) and the design load of the structure. According to Eurocode 3^9^, in the case of elastic analysis, structures are sensitive to second-order effects if the critical load multiplier does not reach the value of 10^10^. Studying Eurocode 3, we get the impression that this requirement applies mainly to frames because the procedure for calculating the critical factor is given in the standard for this type of structure. The Eurocode does not specify a separate procedure for calculating the factor for other structures, such as domes.

Due to the complexity of the problem of designing dome structures, they are the subject of many scientific studies. The analyses concern the choice of the computational model, node stiffness, shape optimization or a combination of bracing to ensure the system's stability^11^. Advanced computer programs, based mainly on the Finite Element Method (FEM), are usually used to analyze the domes. One example is a paper by Chacko et al.^12^, where ANSYS software modeled a ribbed spherical dome with rigid joints. In Ameen^13^, the same software was used to investigate the finite element analysis for the large concrete dome. Another software used to analyze dome structure is ABAQUS, used in Zeinoddini et al.^14^ to study the global stability of an innovative dome comprising double-layer space frame sections and curved flexural members. The paper by Merilmol et al.^15^ analyzed Schwedler's spherical dome with rigid joints.

The issues of creating the dome's geometry are also analyzed, and this problem is mainly related to geodesic domes. A paper by Pilarska and Maleska^16^ presents two different methods for the triangle subdivision to design geodesic domes under seismic analysis. The geodesic dome was also the subject of the paper by Pilarska et al.^17^, in which authors developed an algorithm for selecting the type of structural layout and the reference parameters regarding the technological, strength and weight characteristics. In contrast, the article by Sałapa and Jaworski^18^ compares different kinds of parabolic steel domes with a diameter of 46 m and a height of 8 m. Moreover, the paper used two connection types between structural elements (pin and fix).

Computational models showed that depending on their dimensions and member connectivity, in conjunction with material and geometrical non-linearities (due to imperfections), these structures under combined conditions—mainly dead, snow and wind loads—may exhibit numerous different progressive collapses and various local and global instability phenomena^19–21^. Hence, paper^20^ proved that a non-linear finite element procedure is necessary. Another study^22^ analyzes the impact of earthquakes on geodesic domes designed based on a regular octahedron using two different methods to create their topology. In this work, the authors demonstrated the influence of seismic vibrations on the structures of geodesic domes, depending on the method used to shape their topology.Another issue addressed in scientific publications is the minimization of cross-sections and optimization of the structure, which has also been discussed in numerous articles. In paper^23^, Jadhav and Patil studied the optimization problem of constructing double-layered domes with different span-to-height ratios and supporting conditions. Grzywiński^24^ reviewed layer optimization braced domes, where a mass reduction reached 46.6%. Various methods have been developed or adopted to optimize the structure, indicating the importance of the problem^25–28^. Sometimes the methods of structural optimization can be quite surprising, as in paper Kaveh at all^29^ where the Forensic-Based Investigation algorithm, which is a criminal investigation process in criminology, was utilized. In another publication^30^ the same author utilizes chaotic algorithms for structural optimization.

The paper presents a comparative analysis of steel domes using two design approaches: traditional first-order analysis and an advanced second-order analysis. Although there is much research on the design of domes, it is still challenging to find any standards regarding the choice of design method, as well as the impact of this method on the results obtained. Despite the increasing popularity of these structures, there is a lack of up-to-date research on a comprehensive approach to dome design, not only in public and industrial buildings but also in residential construction^31^. An additional problem is using advanced computer programs for modeling or structural performance of the domes. This research checks the possibility of using the Autodesk Robot Structural Analysis Professional 2022 (ARSAP) software, commonly used in engineering, to model the ribbed dome structure. A critical aspect is the utilization of this particular software, as it is well-known and widely used in the design of bar structures. However, it appears that the software's advanced capabilities are often disregarded, leading to inefficient design of such structures. To substantiate the above assertions, eight models were created, differing in the stiffness of the joints between the main structural elements and the use or lack of bracing. Then, each model was calculated assuming the first linear statics and using a non-linear analysis that considered the second-order effects and imperfections. Analysis results were generated through internal forces, displacements and stresses. Based on the results, conclusions were formulated, confirming the thesis that the ARSAP program is a tool enabling advanced structure analysis. The research presented here illuminates the influence of the design approach on the structural performance of ribbed domes, thereby possessing the potential to propel advancements in dome design and engineering and to promote the widespread adoption of that kind of structure.

## Material and methods

**Figure 1:**
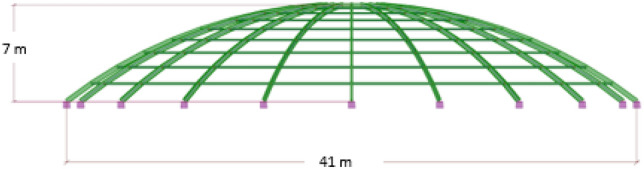
The analyzed object with the structure elements dimensions.

The subject of this article is the analysis of the ribbed dome, consisting of 20 ribs spaced every 18°—meridians, connected by a top ring. There are circumferential purlins between the meridians—parallels on which the roof panels are based. The structure on which this dome is inspired is known as the "Blue Dome" and is the roofing of the main pool at a swimming pool complex in Vichy, France. The dome's diameter is D = 41 m, and the highest height is H = 7 m. The analyzed object with its main dimensions is presented in Fig. 1.

The dome has a parabolic shape. IPE I-sections were used for the meridians, HEA I-sections for the parallels, and C 280 U-sections for the upper ring. Twenty ribs spaced every 18° were designed. Meridians are fixed to the foundation. All sections are made of steel S355. This material is characterized by the following physical properties: young modulus E = 210 GPa, Poisson ratio 0,3, shear modulus G = 81 GPa, and mechanical properties: yield strength 355 MPa, reduction factor for shear 1,54 and limit strength for tension 470 MPa.

The sheathing of the dome was made of single-chamber composite glass panels with selective glass (Effector Sun Effect solar panels). Due to the type of sheathing, the maximum horizontal displacement of the structure was assumed: H/150 = 4.67 cm and the maximum vertical displacement: D/300 = 13.67 cm.

**Figure 2:**
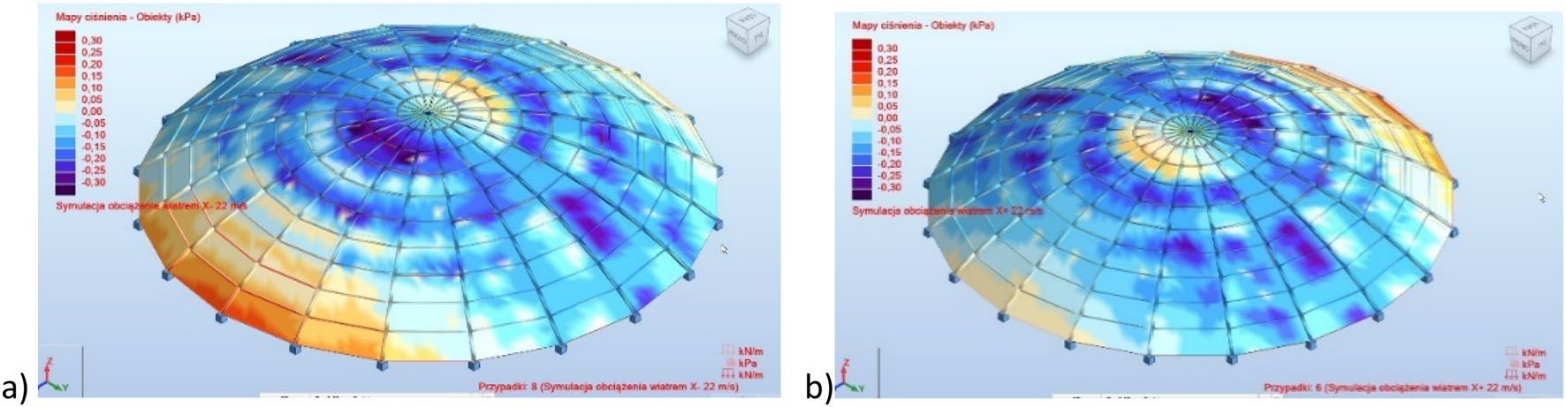
Wind load on the analyzed structure—two selected cases, (**a**) direction " − X" representing the west wind, (**b**) direction " + X" representing the east wind.

When calculating the dome, external impacts were taken into account in the form of permanent structural loads (self-weight of steel elements) and non-structural loads—glass panels—1.4 kN/m^2^, snow loads on the second snow zone in Poland according to Eurocode 1, and the impact of wind with a speed of 22 m/s, in the form of loads automatically generated in the ARSAP 2022 calculation program (Fig. 2).

The computational model of the structure includes two ways of connecting meridians with parallels in the form of pin and fixed node connections. The structure was modeled within a three-dimensional coordinate system, providing six degrees of freedom (three translations and three rotations relative to each X, Y and Z axe). All degrees of freedom are blocked in rigid connections, whereas rotation about the local Y and Z axes is allowed in pinned connections.

**Figure 3:**
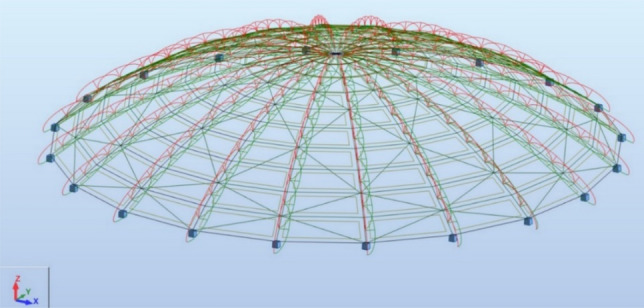
View of the analyzed ribbed dome, taking into account the bow imperfections.

In the first stage, a first-order analysis was performed based on which cross-sections of structural elements were selected. These calculations used automatic load combinations (all possible combinations were considered following Eurocode 0). Then, those load combinations that cause the largest values of internal forces in structural elements and the largest displacements of structure nodes were selected. Subsequent calculations were performed on such selected combinations of loads. Then, a buckling analysis was performed by checking the value of the critical index. After receiving the result indicating the need to complete a second-order analysis, the calculations were repeated considering the non-linear analysis (material non-linearity + sway effect P-Δ) and local imperfections for meridian elements. The model, considering bow (local) imperfections, is shown in Fig. 3.

**Figure 4:**
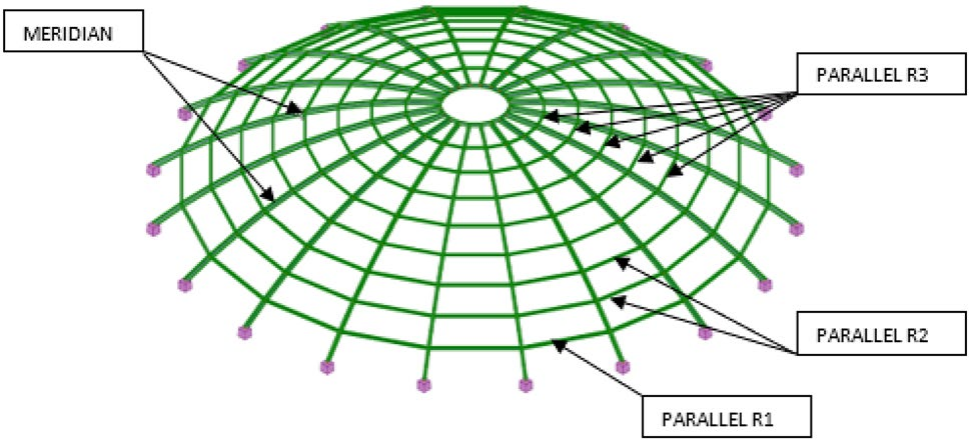
The analyzed object with visible components of the computational model.

The procedure described above was applied to the dome without bracing and to the dome with the adopted bracing system (see Fig. 3), intended as flaccid bars carrying only tensile loads. To rationally dimension the structural elements, they were divided into four groups: meridians, R1 parallels, R2 parallels and R3 parallels (and possibly braces), according to the diagram shown in Fig. 4. The cross-sections of each group of bars were selected so that after meeting the conditions ULS and SLS their mass was as small as possible.

Calculations were made using the Autodesk Robot Structural Analysis Professional 2022 (ARSAP) program. Where feasible, caution was exercised to refrain from altering the default program settings to evaluate the depth of user involvement required to achieve accurate calculations. ARSAP employs the Finite Element Method to calculate internal forces and stresses. In the study, a standard grid size was adopted. For bar elements, the grid is generated by dividing each element into five parts, with each part not exceeding 1 m in length. If the elements are longer than 1 m, the program divides the structural element into more finite elements.

Non-linear analysis is conducted using the incremental method. In this method, the load vector is segmented into *n* equal parts, referred to as increments. Subsequent load increments are applied to the structure only after equilibrium is attained for the preceding increment. A modification during the structural modeling stage involved updating the stiffness matrix after each load subdivision and iteration, thus implementing the Newton–Raphson method. This adjustment ensured the convergence of the calculation process^32^. ARSAP automatically assumes rigid connections between members, i.e. compatibility of displacements and rotations is ensured for all members converging at a given node. These connections have been modified at the modeling stage by release options, where it is possible to release the displacement or rotation of one member relative to another at a node. The method of assuming the releases is described above. By Eurocode 3^33^, to obtain a complete 2nd-order analysis of the structure, it is necessary to adopt bow (local) imperfections. Such imperfections were incorporated into the structure using a specialized function within ARSAP. This function introduces imperfections halfway along the length of a single member, resulting in alterations to the member's geometry by generating a design element representing the deformed shape^32^.

The results of the analysis were divided according to the adopted eight computational models of domes, including the absence or presence of bracings, different ways of connecting between meridians and parallels and analysis type.

1. without bracing, hinge connections, 1st order analysis
2. without bracing, hinge connections, 2nd order analysis
3. without bracing, fix connections, 1st order analysis
4. without bracing, fix connections, 2nd order analysis
5. with bracing, hinge connections, 1st order analysis
6. with bracing, hinge connections, 2nd order analysis
7. with bracing, fix connections, 1st order analysis
8. with bracing, fix connections, 2nd order analysis

The results obtained for each model were compared regarding internal forces, displacements, and stress distribution.

## Results

### Internal forces

**Figure 5:**
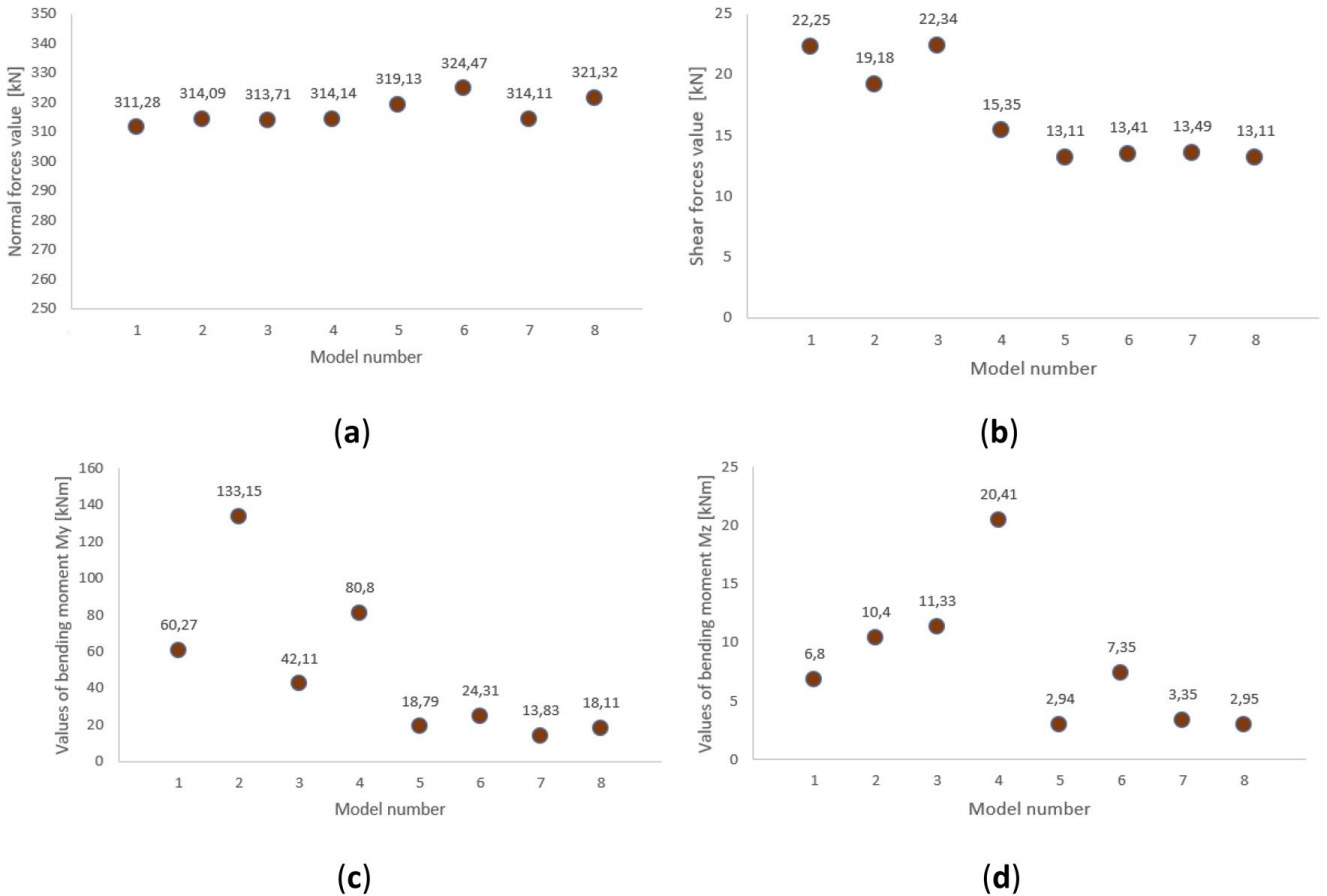
Maximum value of internal forces in every analyzed case: (**a**) normal forces; (**b**) shear forces; (**c**) bending moment M_y_; (**d**) bending moment M_z_.

The maximum values of internal forces that occur in the meridians of the structure are shown in the diagrams in Fig. 5. Axial forces, shear forces and bending moments relative to the y and z axes are presented there.

In all models, comparable values of normal forces were obtained. However, the values of shear forces and moments differ depending on whether the structure is braced or not and the type of analysis performed. The most significant differences occur in the case of the bending moments, where the moments increase significantly in the second-order analysis, especially in structures without bracing. When calculating the structure with pinned nodes without bracing, the program generated an error indicating that the critical load value of the crucial force for axial compression was exceeded. This error also occurred after increasing the meridian cross-section to IPE 550. The analysis results were generated. However, limited confidence should be placed in the accuracy of the obtained results. The study included this case to demonstrate that calculations performed with first-order analysis showed no error. In contrast, analysis of the same structure accounting for second-order effects revealed an exceeding of the critical load. It should also be noted that in the second-order analysis, the size of the internal forces depends on the size of the displacements, i.e., the assumed cross-sections. The internal force that increases the most is the bending moment about the y-axis.

**Figure 6:**
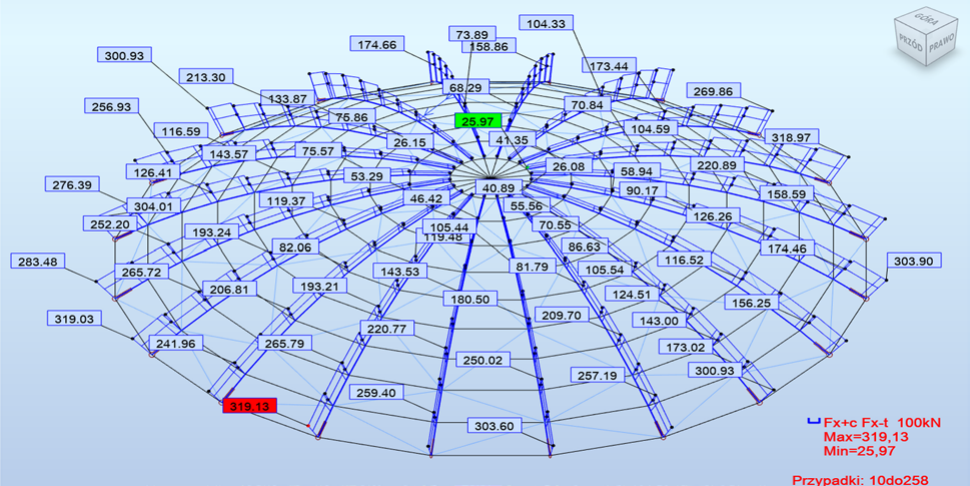
Example of F_x_ force diagrams: braced structure with articulated joints.

**Figure 7:**
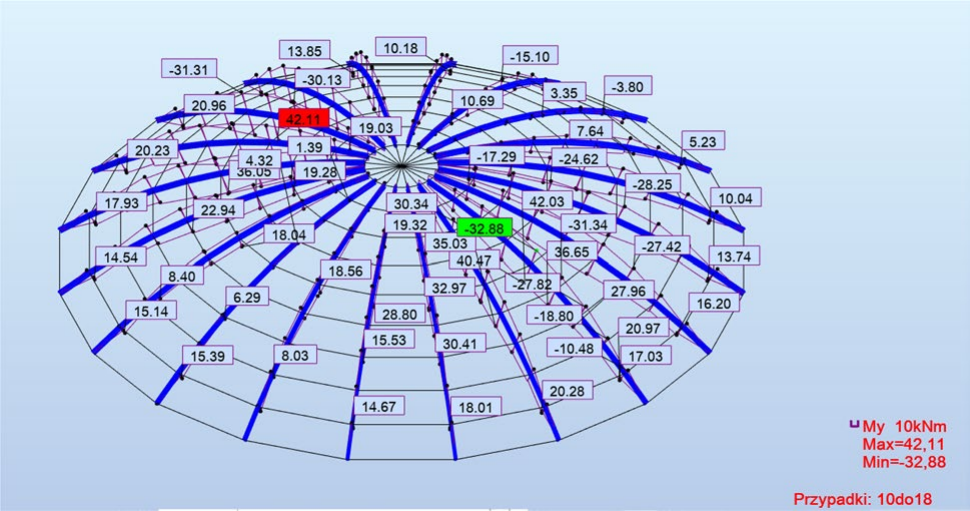
Example graphs of moments M_y_; structure with rigid nodes, without bracing.

The internal force diagrams exhibited similar characteristics in all analyzed cases but had varying values, as illustrated in Fig. 5. Therefore, exemplary internal force diagrams are below in Figs. 6 and 7.

### Critical factor

**Figure 8:**
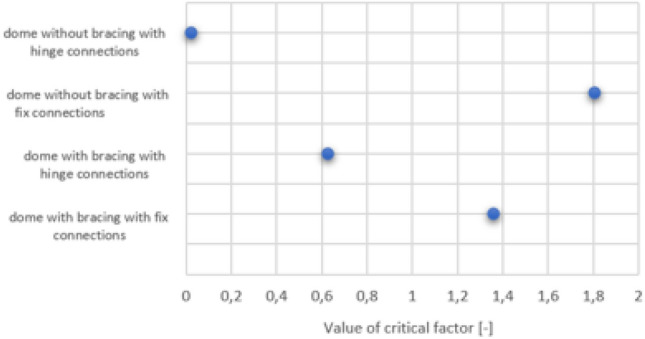
Critical factor value for every analyzed model structure.

For the analyzed structure, a buckling analysis was carried out, and the values of the critical multiplier were generated, which are shown in Fig. 8. For each of the analyzed structures, the value of the multiplier was lower than 3, which, following Eurocode 3, indicates the need to perform a full second-order analysis^34^. It can be noticed that the multiplier values are lower for structures with pinned joints. A shallow value was obtained for structures without bracing. The buckling analysis was performed for cross-sections selected based on the results of the first-order analysis. Therefore, the critical multiplier for a braced structure with rigid nodes is lower than for an unbraced structure, which could indicate a lower stiffness. However, the size of the adopted cross-sections should be considered here, which, in the case of unbraced structures, are larger, according to Tables 1 and 2.

**Table 1:** Stress analysis (structure is shown in Fig. 8).

	Normal min. [MPa]	Normal max. [MPa]	τ_xy_ max. [MPa]	τ_xz_ max. [MPa]	von Mises max. [MPa]
Case	11: SGN/150 = 1*1.15 + 2*1.15 + 4*1.50				
Extreme value	− 187.81	234.04	16.24	26.15	234.04
Section position	0.00	0.00	1.00	0.00	0.00
Members	422	332	255	354	332

**Table 2:** Stress analysis (structure is shown in Fig. 9).

	Normal min. [MPa]	Normal max. [MPa]	τ_xy_ max. [MPa]	τ_xz_ max. [MPa]	von Mises max. [MPa]
Case	11: SGN/150 = 1*1.15 + 2*1.15 + 4*1.50				
Extreme value	− 190.13	220.06	16.92	23.21	220.06
Section position	0.00	0.00	0.24	0.00	0.00
Members	271	271	46	270	271

### Displacements and deflections

**Figure 9:**
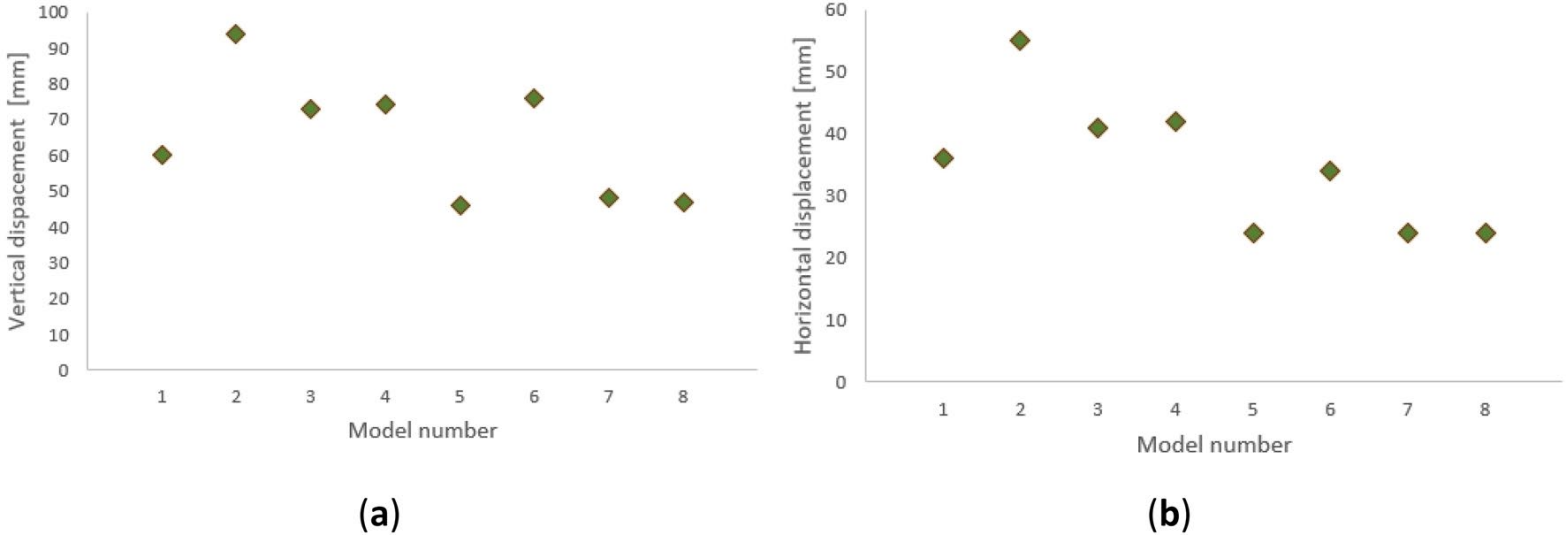
Maximum value of global displacement of the structure: (**a**) vertical; (**b**) horizontal.

The diagrams in Fig. 9 show the size of the horizontal and vertical displacements of the nodes of the analyzed structure. On their basis, it is possible to determine the tendencies of the structure's behavior. We can see that the displacements of structures with pinned joints (models 1, 2, 5, and 6) strictly depend on the type of analysis performed and are larger for the second-order analysis. However, in the case of structures with rigid nodes, the type of analysis slightly affects the size of the displacements obtained.

**Figure 10:**
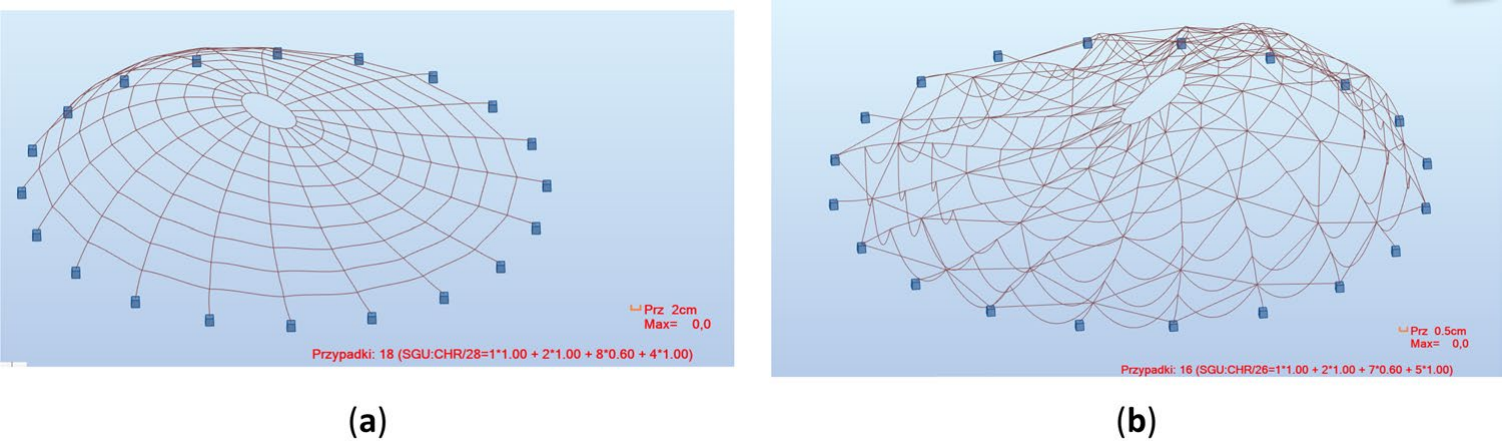
Example of structure deformations: (**a**) without bracing, with fixed connections; (**b**) with bracing, with hinge connections.

**Figure 11:**
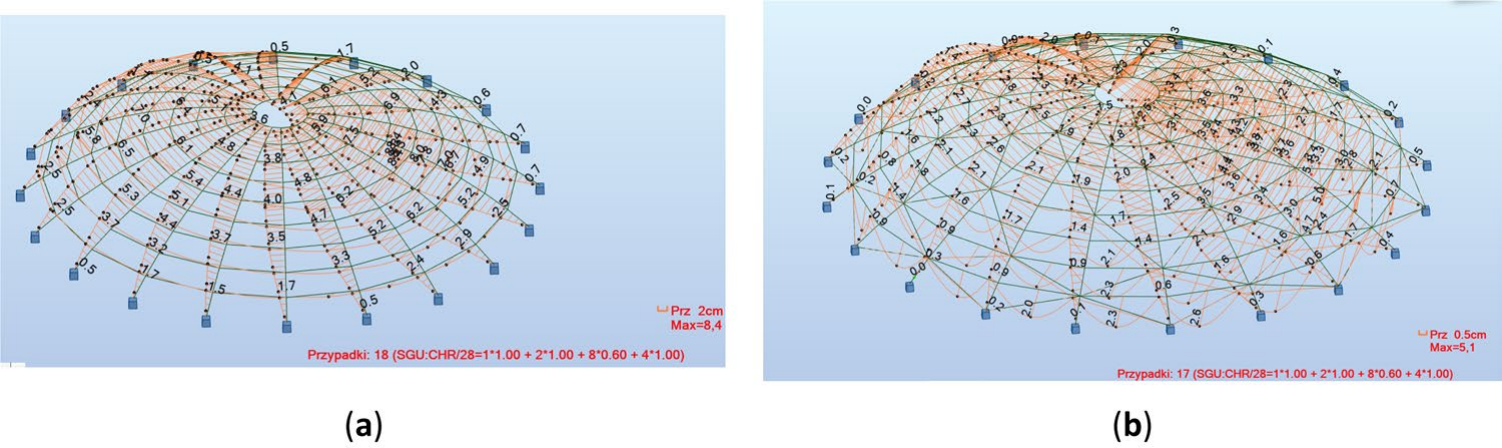
Example of joint displacements: (**a**) without bracing, with fixed connections; (**b**) with bracing, with hinge connections.

The structure with rigid nodes also differs in the form of deformation. The stiffness of the connections between the meridians and parallels significantly stabilizes the beam elements (parallels), which causes more significant displacements on the meridians. In the case of pinned joints, the main displacements are on parallels. Chosen examples of deformation of both types of structures are shown in Figs. 10 and 11. In the figures, deformations for various load combinations are presented to illustrate the nature of the phenomenon. Cases with the highest absolute values of horizontal displacement were selected. The greatest deformations of the structure occur when strong winds combine with snow loading.

### Stress analysis

Stress analysis was carried out each time for the analyzed model. Calculations were made for a structure dimensioned according to a given type of analysis following Tables 1 and 2. Reduced stresses (von Mises stresses) and Normal stresses (relative to the element's x-axis) were considered in the analysis. Reduced stresses were used to determine the structure's general condition; the yield point was assumed as the allowable value of these stresses. In no case did the structure plasticize. The stress distribution varies depending on the type of structure and reflects the adopted support assumptions for individual structural elements.

**Figure 12:**
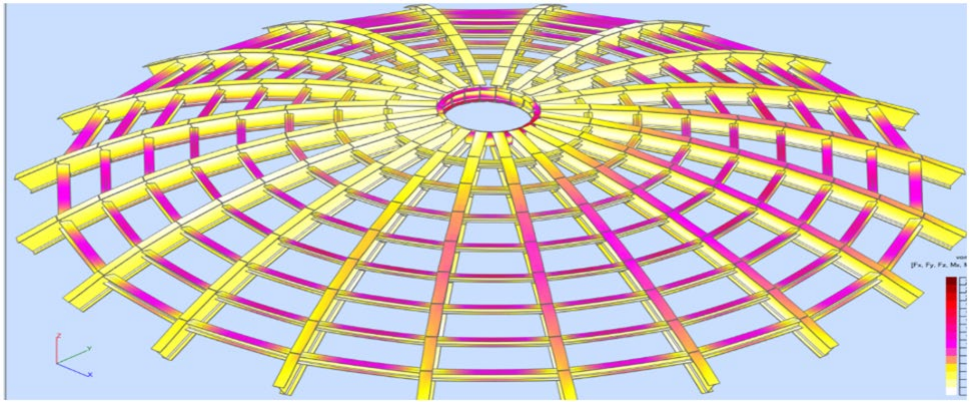
Stress analysis of the structure—model 2 (pin connections, second-order analysis)—reduced stresses.

**Figure 13:**
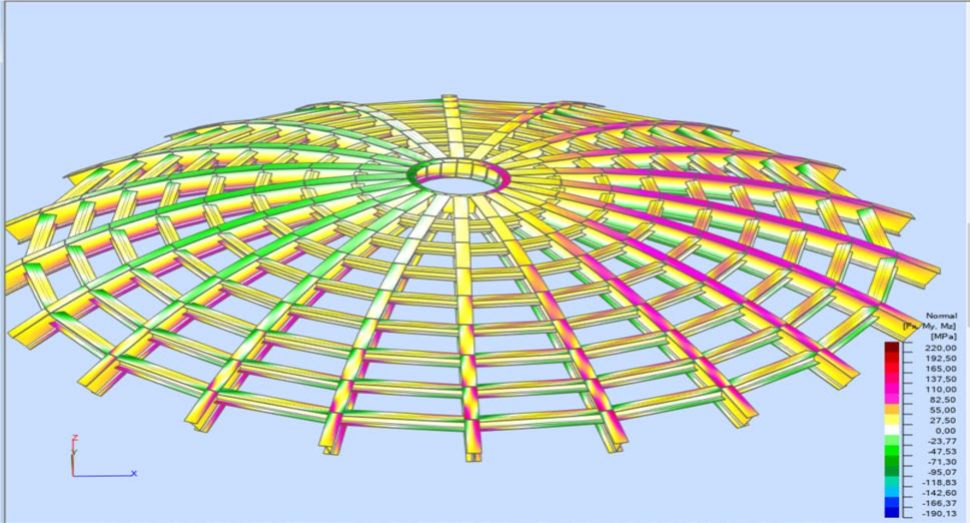
Stress analysis of the structure—model 4 (fix connections, second-order analysis)—normal stresses.

**Figure 14:**
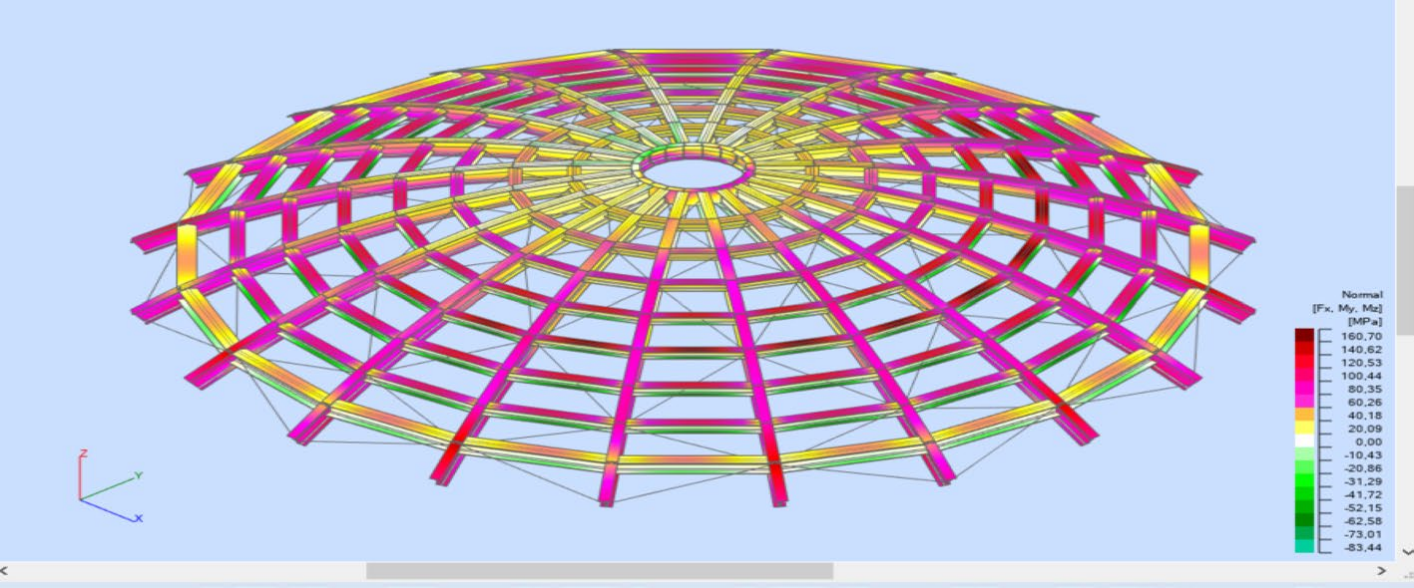
Stress analysis of the structure—model 5 (pin connections, 1st order analysis)—normal stresses.

**Figure 15:**
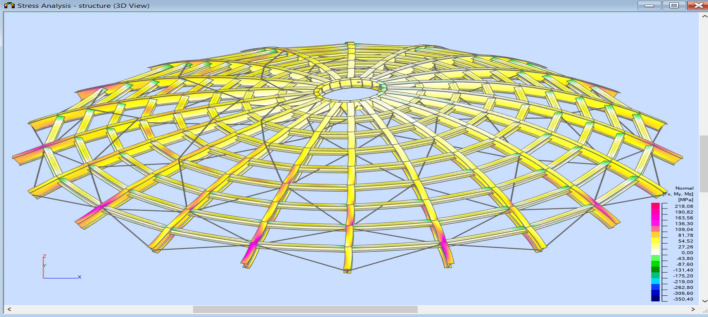
Stress analysis of the structure—model 8 (fix connections, second-order analysis)—normal stresses.

In the case of a braced structure, an evident influence of adopting the bracing system on the main elements (meridians) can be observed. Every second meridian shows lower stresses in the support zone. In addition, the structure is fastened in each case, and stress reduction in the support zone is visible in the middle part. Selected stress analysis results are presented in Figs. 12, 13, 14, 15 and Tables 1, 2, 3, 4 below.

**Table 3:** Stress analysis (structure is shown in Fig. 10).

	Normal min. [MPa]	Normal max. [MPa]	τ_xy_ max. [MPa]	τ_xz_ max. [MPa]	von Mises max. [MPa]
Case	159: SGN/150 = 1*1.15 + 2*1.15 + 4*1.50				
Extreme value	− 83.44	160.71	6.48	24.90	160.71
Section position	0.5	0.5	0.00	0.0	0.5
Members	354	327	296	336	327

**Table 4:** Stress analysis (structure is shown in Fig. 11).

	Normal min. [MPa]	Normal max. [MPa]	τ_xy_ max. [MPa]	τ_xz_ max. [MPa]	von Mises max. [MPa]
Case	12: SGN/151 = 1*1.15 + 2*1.15 + 5*1.50				
Extreme value	− 350.40	218.08	8.99	25.92	350.40
Section position	1.0	0.0	0.76	0.00	1.00
Members	706	182	246	371	706

### Cross-section design—limit states of the structures

The procedure for selecting the cross-sections of structural elements was carried out for the analyzed models following Eurocode 3. Tables 5 and 6 below present the results of the dimensioning of structural elements. Table 5 applies to models 1–4, i.e., unbraced structures, while Table 6 applies to models 5–8, i.e. braced structures. In the case of an unbraced structure, the condition resulting from the serviceability limit state (SLS) was decisive in selecting such a section. In contrast, in the case of a braced structure, it was the condition of the ultimate limit state (ULS).

**Table 5:** Results of the analysis—structure without bracing.

Model number/structure description	1/ Hinge—1st order analysis		2/ Hinge—second-order analysis		3/fix—first-order analysis		4/fix—second-order analysis	
Limit state	ULS	SLS	ULS	SLS	ULS	SLS	ULS	SLS
Selected cross-section of meridians	IPE 240	IPE 360	IPE 300	IPE 400	IPE 240	IPE 300	IPE 300	IPE 330
Selected cross-sections of parallels R1	HEA 160	HEA 160	HEA 180	HEA 180	HEA 160	HEA 160	HEA 180	HEA 180
Selected cross-sections of parallels R2	HEA140	HEA140	HEA 140	HEA140	HEA140	HEA 140	HEA 160	HEA 160
Selected cross-sections of parallels R3	HEA 120	HEA 120	HEA 120	HEA 120	HEA 120	HEA 120	HEA 140	HEA 140
Bracing	–	–	–	–	–	–	–	–
Decisive condition	SLS		SLS		SLS		SLS

**Table 6:** Results of the analysis—construction with bracing.

Model number/structure description	5/ Hinge—1st order analysis		6/ Hinge—second-order analysis		7/fix—1st order analysis		8/fix—second-order analysis	
limit state	ULS	SLS	ULS	SLS	ULS	SLS	ULS	SLS
Selected cross-section of meridians	IPE 220	IPE 220	IPE 220	IPE 240	IPE 220	IPE 220	IPE 220	IPE 220
Selected cross-sections of parallels R1	HEA 120	HEA 120	HEA 120	HEA 120	HEA 120	HEA 120	HEA 120	HEA 120
Selected cross-sections of parallels R2	HEA120	HEA120	HEA120	HEA 120	HEA 120	HEA 120	HEA 120	HEA 120
Selected cross-sections of parallels R3	HEA 120	HEA 120	HEA 120	HEA 140	HEA 120	HEA 120	HEA 120	HEA 120
Bracing	φ 14	φ 14	φ 16	φ 16	φ 12	φ 12	φ 14	φ 14
Decisive condition	ULS		ULS		ULS		ULS	

The primary condition affecting the size of the cross-section of the meridians is the interaction of the axial force and the bending moment, considering the buckling about the y-axis. At the same time, in the case of parallels, it is the interaction of the axial force and the bending moment considering the buckling about the z-axis.

**Figure 16:**
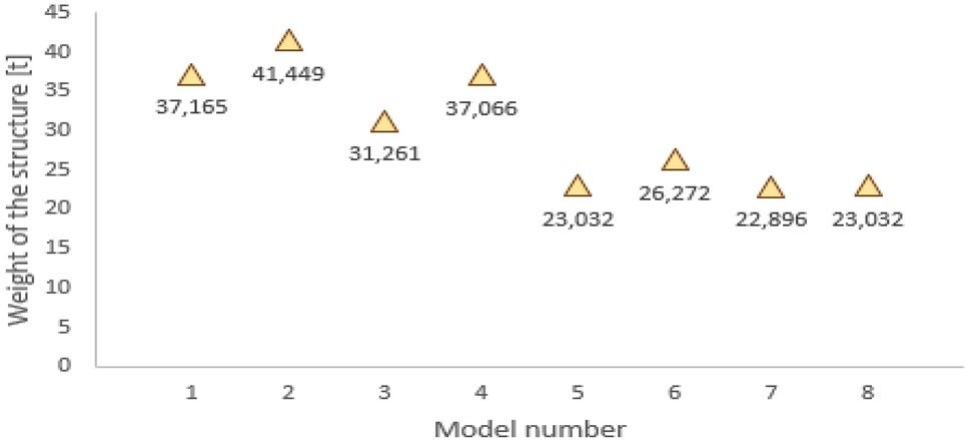
Comparison of structure weights for various construction solutions of the dome.

Figure 16 contains a comparison of the masses of the bar structure of the domes for various construction solutions, which is of particular interest to us. Calculations using full second-order analysis show that the smallest mass of the structure occurs with a braced structure, with rigid connections of meridians with parallels. The difference between the weight of the sections in model 2 and model 8 is about 45%.

## Discussion

The dome structure offers numerous advantages, including structural efficiency, strength, durability and ease of maintenance. These qualities have contributed to its rising popularity in industrial and residential construction^31^. Dome structures can be designed using steel, wood, brick, or innovative materials. There have even been efforts to utilize 3D printers to print dome structures^35^. Despite this, scientific articles remain scarce addressing an effective method for modeling this type of structure. Only a few recent scientific papers provide insight into the potential of parametrization in the design of ribbed domes^30^ or demonstrate the effectiveness of using different materials to design domes^36^. Current advanced computer programs, mainly utilizing the Finite Element Method, allow for the modeling of such structures. However, their usage requires advanced knowledge and skills, making them less user-friendly for the average designer.

The influence of wind and snow loads on the structural behavior of the ribbed dome is a critical aspect of its analysis. The dome was subjected to various external impacts, including snow loads based on Eurocode 1 for the second snow zone in Poland and wind loads with a speed of 22 m/s. The wind load analysis considered different wind directions, such as west and east winds, to assess the structure's response under varying conditions. The computational model incorporated two types of connections between meridians and parallels: pin and fixed node connections. The analysis revealed that the presence or absence of bracing significantly affected internal forces, displacements, and stress distribution within the structure. Notably, the study highlighted that structures without bracing exhibited higher displacements, emphasizing the importance of bracing systems in enhancing stability. Additionally, the research emphasized the necessity of considering second-order effects in structural calculations to ensure accurate and reliable results, especially in determining cross-sections and critical factors for structural elements. The comprehensive analysis provided insights into how wind and snow loads impact the structural behavior of ribbed domes, underscoring the importance of these considerations in designing resilient and efficient structures.

The calculations that have been carried out above show that an available and widely used program supporting the structure design process, such as ARSAP, can be used to obtain the results of advanced computational analysis. This program is based on the Finite Element Method, which is a simplified method, and the results depend directly on the skills and understanding of the program by the designer^37^. In the above example, the default functions of the program were used, and the method of approximation of differential equations and the size and shape of finite elements were not changed. Therefore, the obtained results may show certain inaccuracies, which were also not estimated in this work.

The presented results represent the initial phase of the research. In the subsequent steps, further analysis of the results and more detailed investigations into the influence of various factors on the behavior of the dome structure and the accuracy of results are warranted. It is advisable to repeat the calculations by dividing the structural elements into parts, which would impact the size of the assumed finite elements, and to assess how such a division affects the obtained results. At this stage of the research, the accuracy of the obtained results was assumed based on the knowledge and experience of the article's authors in engineering design. It should be noted that these results are of the same order as the results available in the literature^38,39^. The results have not been verified by any other method or software. Nonetheless, some form of verification should be conducted. It will be done using software dedicated to the Finite Element Method, such as ANSYS or ABAQUS. This would allow us to assess the degree of accuracy of the calculations with the work needed to create the computational model. The methods redefined in this work and will be further validated and refined in subsequent ones, which may contribute to the development of further scientific issues, such as the prestressed steel domes, where initial research efforts were undertaken^40^ or suspend-dome^41,42^. It should be noted that in these cases, the software used also provides specific capabilities that should be further tested and verified.

## Conclusions

The ARSAP 2022 program is suitable for analyzing commercial projects' dome structures. ARSAP 2022 allows calculations considering the second-order effects in global imperfections (sway with the P-Δ effect) and local imperfections. In each of the analyzed cases, the value of the critical multiplier was determined; in each case, this value was lower than 3. According to Eurocode 3, it is necessary to consider the second-order effects when calculating the structure. Critical factor values are higher for rigid nodes, which correspond with higher stiffness of the whole system. In the case of structures without bracing, the analysis taking into account the second-order effects did not allow the adoption of values similar to those of the cross-sections of structural elements as the first-order analysis. The cross-sections of meridians have increased to a greater extent, and the cross-sections of parallels have decreased to a lesser extent. The following permissible displacement values were adopted: vertical D/300 and horizontal H/150. It was noticed that the displacement values of the structure nodes depend mainly on the stiffness of the meridians. In the case of a less stable structure—a dome without bracing—the selection of cross-sections was determined by the SLS condition. For a more stable structure—a dome with bracings—the difference between the meridian cross-sections selected from the SLS and ULS condition did not occur or was insignificant. The primary condition determining the selection of cross-sections in the ULS for meridional elements is the interaction of the axial force and the bending moment, considering the y-axis buckling.

In contrast, it is the interaction of the axial force and the bending moment for latitudinal elements, considering the z-axis buckling. Results are the same in all analyzed cases. In the braced structure, taking into account the effects of the second order caused a slight increase in internal forces, which most often did not increase the cross-sections of structural elements—meridians and parallels. Only the cross-sections of the bracings were increased, which was caused by higher axial forces in the bracings. In the case of an unbraced structure, the choice of the connection method at the nodes significantly impacts the magnitude of the internal forces and, thus, the structure's weight. In the case of a braced structure, the choice of the type of connection is less important, which entails the possibility of making pinned connections, which are much simpler in execution.

The study innovates by systematically comparing two design approaches for steel domes—traditional first-order and advanced second-order analyses. This provides valuable information on the effects of different methods on the structural performance of ribbed domes. Moreover, Autodesk Robot Structural Analysis Professional 2022 (ARSAP) software, widely used in engineering, demonstrated the potential of advanced computational tools to model and analyze complex structures such as ribbed domes. This demonstrates the capabilities of modern software in streamlining the design process and optimizing construction performance. The second aspect is that the study performs a comprehensive analysis by considering various factors such as joint stiffness, stiffening, and non-linear effects, including second-order effects and imperfections. This holistic approach provides a deeper understanding of the behavior of ribbed domes under various conditions. By creating eight different models and rigorous analysis, the study validates its findings regarding the impact of the design approach on structural performance. This increases the credibility of the conclusions drawn and the credibility of the research results. The research presented here illuminates the influence of the design approach on the structural performance of ribbed domes, thereby possessing the potential to propel advancements in dome design and engineering and to promote the widespread adoption of that kind of structure. By incorporating imperfections into the computational model, the analysis can more accurately simulate real-world conditions and predict how the structure will behave. Imperfections affect the distribution of internal forces, displacements, and stress patterns within the dome, highlighting areas of potential weakness or vulnerability. Also, imperfections can influence the critical load multiplier, which is a key parameter in assessing structural stability against second-order effects like buckling.

The limitations and assumptions made during the analysis process, including potential inaccuracies in results, are crucial considerations in understanding the reliability of the study on the steel ribbed dome's structural performance. The research acknowledges that the traditional design approach based on first-order elastic analysis may lead to inefficient design time, excessive material consumption, and potential safety risks due to designing close to structural limits. By transitioning to an advanced second-order analysis method that considers structural deformation effects on internal forces, the study aims to address these limitations. However, it is essential to note that the accuracy of results may be affected by simplifications or assumptions made in the computational model. For instance, imperfections introduced into the structure for analysis may not fully capture real-world conditions, potentially leading to discrepancies between simulated and actual behavior. Moreover, using software like Autodesk Robot Structural Analysis Professional 2022 introduces complexities in modeling and analysis, which could impact result accuracy. These factors underscore the need for a critical evaluation of assumptions, model parameters, and software capabilities to ensure the validity and reliability of the analysis outcomes.

Based on the obtained results and discussion, some main concluding remarks can be drawn as follows:

- The analysis results confirm that the ARSAP program facilitates advanced structural analysis. In structures of this type, it is necessary to conduct buckling analysis to assess the need for considering second-order effects in calculations
- The performed calculations demonstrated the necessity of considering second-order effects in all cases.
- The case involving hinge connections without bracing revealed an error in exceeding the critical load, which only occurred with second-order analysis. Based on this, it can be inferred that structural stability analysis is necessary to estimate the requirement for considering second-order effects.
- In the case of an unbraced structure, the selection of cross-sections was influenced by the serviceability limit state (SLS) condition. In contrast, in a braced structure, it was determined by the ultimate limit state (ULS) condition.
- The meridian stiffness was observed to influence the structure nodes' displacement values primarily. In the case of a less stable structure—a dome without bracing—the selection of cross-sections was dictated by the serviceability limit state (SLS) condition
- Comparable values of normal forces were obtained in all models. However, discrepancies in shear forces and moments were observed depending on whether the structure was braced or not and the type of analysis conducted. Significant differences were observed in bending moments, which increased notably in the second-order analysis, particularly in unbraced structures.

A comparison of the masses of the bar structure of the domes for various construction solutions showed notable differences, with variances of approximately 45%.

## Data Availability

The datasets used and/or analysed during the current study available from the corresponding author on reasonable request.
